# Leadership and Conflict Management Style Are Associated with the Effectiveness of School Conflict Management in the Region of Epirus, NW Greece

**DOI:** 10.3390/ejihpe10010034

**Published:** 2020-02-14

**Authors:** Elisavet Chandolia, Sophia Anastasiou

**Affiliations:** 1 Graduate MEd Program Hellenic Open University, 26500 Patra, Greece; std105271@ac.eap.gr; 2 Directorate of Secondary Education of Arta, 47100 Arta, Greece; 3 Faculty of Social Sciences, University of Ioannina, 45500 Ioannina, Greece

**Keywords:** School Leadership, conflict management, teachers

## Abstract

There are few options available for school managers who wish to effectively tackle school conflicts. The aim of the present work was to assess the issue of school conflict, its sources, and the effectiveness of different conflict management styles in Secondary Education school units in Greece. Teachers (n = 128) from twelve randomly selected schools in the region of Epirus, NW Greece, participated in the present work. Teachers’ views on their school Principals’ leadership style as well as the sources, the type(s), and the severity of conflict in their school unit were surveyed. Conflict appeared to be a frequent issue in schools. Frequent sources of conflict included interpersonal and organizational parameters. School leaders exhibited a range of conflict management styles. *Compromise* and *Collaborative* styles were frequently observed, followed by *Smoothing* and *Forcing*. *Avoidance* was less frequently exhibited by school leaders. The *transformational* and *transactional* leadership styles exhibited were equally effective in successful conflict resolution, whereas a *laissez-faire* leadership style was not. The results indicate that leadership and conflict management style can be associated with the effectiveness of conflict management.

## 1. Introduction

School leaders have a significant role in responding to the rising demands of modern society for effective school management [1]. They may adopt different leadership styles, and this can be a critical parameter for the success of school and its leadership [2]. In this context, school Principals are a key parameter in ensuring growth, goal achievements, and corporate success [3]. School leaders are expected to perform complex tasks and act both as managers and leaders. Their role includes: support to teachers, students and parents; liaising with parents and other stakeholders [4]. This range of tasks, complexity and multidimensional role can be described as the *art* of leading [5,6]. The *art* of leadership is crucial for a school to be effective under its particular internal and external school variables, including parents’ and society’ expectations, teachers’ workload and school climate including the culture of change and innovation [6,7,8].

*Leadership* can be described according to several theories and different leadership styles that can be grouped accordingly, including: trait theories, behavioral theories, situational theories, contingency theories, transactional leadership, transformational leadership, empowerment leadership, authentic leadership, strategic leadership, symbolic leadership, servant leadership and innovative leadership. School Principals may exhibit a range of leadership styles according to the national educational systems’ policies, their personality traits, values, experiences and skills, as well as the particular issues and environmental parameters of their school unit [8,9,10,11,12,13,14]. In fact, effective leaders can adapt their leadership style depending on the context and situation of their school unit [8,12,13,15,16,17,18].

The significant role of school leaders in Greece is confirmed by the reported effects of leadership style on school climate, students’ achievements, and teachers’ job satisfaction [8,12,17,18]. 

The Greek educational system is characterized by a highly hierarchical structure with limited opportunities for creativeness and initiatives for school Principals. The Greek Ministry of Education is the main decision-making center responsible for the formulation of key educational policies and control. It decides on almost all the issues concerning the allocation of funds, school operation, and human resources management policies such as teachers’ appointments and transfers. As a result of this top-down hierarchical structure, school leaders in Greece perform in a bureaucratic system with limited power and funds at the school level [4,8,10]. Nevertheless, even in a centralized educational system, school Principals have a decisive role in school management with significant effects on teachers and pupils. School leaders can be creative and inspirational, having an impact on several school parameters. Furthermore, school leaders may be required to perform under far from ideal conditions, to handle difficult situations and explore various ways in resolving conflicts and maximizing the potential outcome of their schools [13,19]. 

One issue that school leaders are expected to handle successfully in their units is the issue of *school conflict*. *Conflict* is a natural phenomenon in organizations as a result of interactions among people. Managers can handle conflict according to their personality skills, organizational settings and the context of the conflict. Successful management intervention may not eliminate conflicts, but is expected that managers will increase the positive rather than the negative outcomes of conflicts in an organization. For example, conflict can lead to the exchange of ideas, modernization and adaptation needed for organizational success. It can also have dramatically negative effects on the workplace, sometimes augmented by conflict-generating parameters such as poor communication and differences in views, values and priorities in an organization [20]. In other words, conflict will always be an issue for management and conflict management can make a difference in terms of the consequences of conflict for the individual as well as the organization [21,22].

Irrespective of the educational system or organizational parameters, conflict may result from differences of opinion, values, and feelings in the workplace [23,24,25,26]. As in any organization, school leaders are expected to handle situations of conflict in their school unit. It is anticipated that school leaders will determine how to address or minimize tensions in their schools based on their training and skills. Conflict in schools can be augmented by personal or organizational parameters [24]. Leaders set the tone for conflict management through their leadership styles. Their work experience and perceptions can moderate their priorities, strategies and style during conflict management [24,25]. 

Leaders may have different leadership styles and different conflict management styles. A review of the relevant literature reveals that there are a few options available for school managers who wish to tackle a school conflict and these can be grouped into five different approaches [21,22,23,24]: *Avoidance, Compromising, Collaborating-Integrating, Forcing-Competing, Smoothing-Accommodating.*

*Avoidance* is a managing conflict style whereby an individual does not succeed in/avoids adequately addressing a conflict but instead postpones, withdraws, or sidesteps. In most cases, individuals will tend to avoid conflict due to fear of getting involved in the dispute or they may lack the confidence in their managing conflict skills [26]. 

*Compromising* is a conflict managing approach aimed at finding a solution that is mutually acceptable and expedient and partially satisfies both the involved parties [21,22,23,24,25,26,27,28]. 

The *Collaborating-Integrating* approach entails making an effort of working with the other individual in searching for a solution that fully addresses the issue at hand, satisfying all the involved parties. It includes identification of the underlying concerns of one’s opponent and finding the alternatives for meeting the interests of each party [21,22,29,30]. 

Adopting a *Forcing-Competing* approach entails pushing one’s opinion at the expense of others and maintaining active resistance to the action of the other person. The *forcing* technique is used in situations whereby one needs to fight for ones’ rights/opinion, resisting pressure or aggression [17,21,22,23,24,31].

The *Smoothing-Accommodating* style accommodates concerns of ‘others first’ instead of giving one’s own interests top priority. The *smoothing* technique is appropriate when it is crucial to provide a relief that is temporal from conflict and/or when the problem is not meaningful to one person compared to others [21,22,32]. 

Several parameters can increase the likelihood of a school conflict. For example, a recent study in Primary Education units in Greece exhibited different possibilities of potential school conflicts according to the level of urbanization and the adoption of a constructive conflict management approach by school leaders [24]. A school leader may not intervene with the teaching process if, for example, the school exhibits significant educational outcomes. On the contrary, in a situation of low students’ performance, a school leader may give priority in the teaching processes or in reducing students’ aggressiveness as a prerequisite for both improving educational outcomes and teachers’ job satisfaction and commitment. School leaders and teachers must find ways to successfully resolve school conflicts. Furthermore, school leaders and teachers are expected to work with students in developing their understanding concerning conflict. 

The role of school leader is also crucial in handling conflicts between teachers or between teachers and parents. A competent leader can identify the conflict management strategy appropriate for each case and recognize which conflict management qualities and skills or solution strategy is most suitable for each situation [32]. Leaders set the tone for conflict management through their leadership styles. 

Successful school leaders are expected to develop their leadership skills, including conflict management skills, which will aid in achieving positive outcomes in their school unit, including effective conflict resolution, ensuring that relationships with the other staff members and/or the school community involved in a conflict are not adversely affected [33]. 

There is some evidence to suggest that certain leadership traits can be associated with particular conflict management styles [20,34]. For example, a *transformational* leader may attempt to inspire teachers in successful conflict resolution. Contrary to transformational leadership, a *laissez-faire* leadership style can be consistent with the *Avoidance* conflict management strategy as passive leadership is characterized by avoiding dealing with a problem. It has been argued that *laissez-faire* leadership will ignore conflicts and this will increase conflicts in the work place, for example, conflicts among co-workers [15,27].

The aim of the present work was to investigate the frequency of school conflicts in relation to the leadership and conflict management style exhibited by school Principals. The hypothesis is that the effectiveness of conflict management will vary according to leadership style and conflict management style.

## 2. Materials and Methods 

The random number generator function of Microsoft excel was used to randomly select twelve (12) Secondary Education school units in the region of Arta, Epirus, NW Greece. Prior to the distribution of the questionnaires, approval from school Principals of the Secondary Education school units that participated in the present research work was obtained, teachers were informed about the purpose of the research, and their consent to participate in the study was obtained. The research protocol was approved as a graduate research project of Mrs Chandolia and was conducted in accordance with the guidelines of the Hellenic Open University, and further approval of the ethics committee was not required. 

Questionnaires (n = 150) were distributed during Spring, 2019. The return rate was 85.33% (n = 128). This sample corresponds to 26.66% of teachers and 36.36% of school units in the region. 

The first part of the questionnaire included general questions related to demographic and personal factors such as: gender, age, marital status, teaching experience, etc. 

Additionally, teachers’ views on the leadership style of their school Principals as well as the sources, type(s), and severity of conflict in their school unit were surveyed using previously used questionnaires adopted and validated for use in Greece. 

### 2.1. Sources and Frequency of Conflict and Conflict Management Style

Teachers’ perceptions regarding the frequency (7 items) and sources (7 items) of school conflict and conflict management style (10 items) were surveyed using a questionnaire previously used in relevant research in Greek schools that was reported to provide satisfactory internal consistency and reliability [24,26]. The questionnaire was used to measure five conflict management modes: *Avoiding*, *Compromising*, *Collaborating*, *Forcing-Competing*, and *Smoothing-Accommodating*. Teachers were asked to respond using a five point Likert scale: (0 = never, 1 = rarely, 2 = sometimes, 3 = often and 4 = very often).

### 2.2. Leadership Style

The Multifactor Leadership Questionnaire (MLQ, 5X-Short) of Bass et al. [27], adapted into the Greek language by Magoulianitis [29] was used so as to assess transformational and transactional leadership behavior. MLQ’s five transformational, three transactional, one laissez-faire, and the three outcome scales were included: (i) The transformational scales: inspirational motivation, idealized influence (behaviors), inspirational motivation, intellectual stimulation, and individualized consideration, (ii) the transactional scales: contingent reward, management-by-exception, and management-by-exception passive, (iii) laissez-faire is used as a non-leadership contrast to transformational and transactional leadership approaches, and (iv) the three outcome criteria: followers’ extra effort, the effectiveness of leader’s behavior, and followers’ satisfaction with their leader. 

The questionnaire contained 39 items which were used to assesses the presence or absence of various leadership styles using a five points scale (0 = never; 1 = rarely; 2 = sometimes, 3 = often; 4 = very often). Data were analyzed with SPSS (version 14.01), normality tests (Shapiro Wilk test) were performed to confirm if data were normally distributed, and significance of differences was evaluated with one-way analysis of variance (ANOVA) followed by Tukey’s post-hoc tests.

## 3. Results

The demographic characteristics of the teachers who participated in the present work are presented in Table 1. Most of the teachers were above 40 years-old and about half of them had professional experience of over 16 years serving in their school unit. 

The descriptive statistics and Cronbach’s reliability for the transformational and transactional components of the MLQ are presented in Table 2. The rating of School Principals ranged between 3.05 and 3.54 for the transformational leadership style and between 2.63 and 3.55 for the transactional leadership style, exhibiting high scores compared to the laissez-faire leadership. 

Tukey’s honestly significant difference (HSD) post-hoc tests showed collective preferences towards: Idealized influence (attributed); idealized influence (behavior); inspirational motivation; intellectual stimulation; individualized consideration; contingent reward, and management-by-exception (active). The least frequently perceived leadership traits were management-by-exception (passive) and laissez-faire leadership.

The presence of different leadership styles was assessed according to the scores of teachers’ answers (0 = never; 1 = rarely; 2 = sometimes, 3 = often; 4 = frequently). A score above 3 indicates that a particular leadership style was *often* or *frequently* exhibited. A score between 2 and 3 indicates that a particular leadership trait was *sometimes* used.

### 3.1. Analysis of the Leadership 

**Figure 1:**
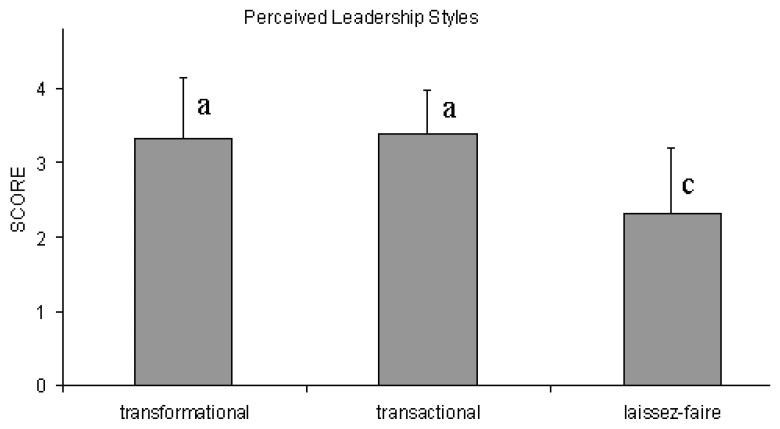
Teachers’ perceptions of the leadership style of their Principal.

Teachers’ perceptions of their Principal’s leadership style (Figure 1) indicate that their Principal frequently used transformational or transactional leadership styles, whereas a passive leadership style was less frequently exhibited (Tukey’s HSD post-hoc tests, *p* < 0.01). 

In order to examine differences by school size, two groups were established on the basis of the distribution of the data and the effect of school size on the leadership style exhibited was investigated by comparing “small school units” with “large school units”. “Small” group of schools included units with less than 11 teachers serving, which is about three times smaller than the national average for schools in Greece. Schools with about triple the number of teachers were considered as “large” school units in the present work. 

A statistical comparison between the leadership style of “small” and “large” school units is presented in Table 3. Compared to small school units, larger schools scored significantly higher (t-test, *p* < 0.05) on the transformational leadership style. The reverse was exhibited on the laissez-faire leadership style, where small schools exhibited significantly higher (*p* < 0.05) scores. There was no significant difference between small and large school units in the scores of transactional leadership styles. 

### 3.2. Frequency of Conflicts

**Figure 2:**
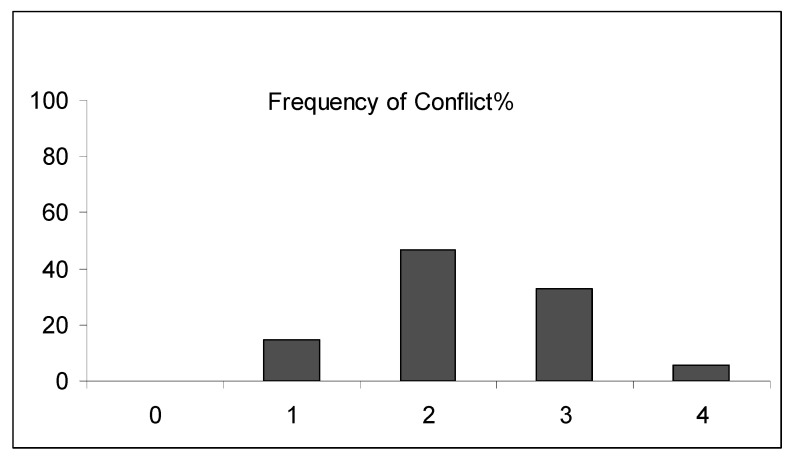
Teachers’ perceptions of the frequency of conflicts exhibited in their school units.

Teachers’ perceptions on the frequency of conflicts in their school units are presented in Figure 2. The frequency of conflict occurring in schools was assessed according to the scores of teachers’ answers (0 = never; 1 = rarely; 2 = sometimes, 3 = often; 4 = frequently). A score above 3 indicates an *often/frequent* occurrence of school conflict. A significant number of teachers (38.28%) reported an *often/frequent* occurrence of conflict in their school.

### 3.3. Sources of School Conflicts

**Figure 3:**
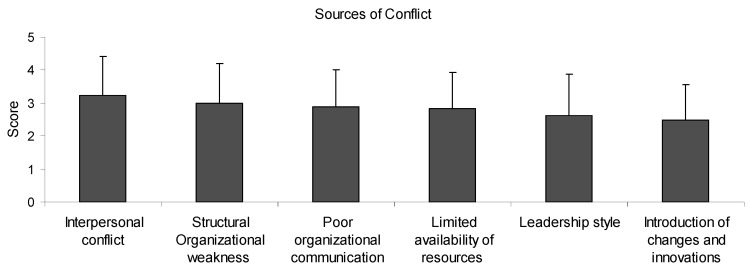
Teachers’ perceptions of the sources of conflict exhibited in their school units.

Teachers’ perceptions on the sources of school conflict was assessed according to the scores of their answers (0 = never; 1 = rarely; 2 = sometimes, 3 = often; 4 = frequently) on a range of possible sources: interpersonal conflict; structural organizational weakness; poor organizational communication; limited availability of resources; introduction of changes and innovations; leadership style (Figure 3).

A score above 3 indicates a high likelihood of a particular source of conflict compared to a score between 2 and 3, which indicates a low likelihood of a particular source of conflict. 

There was no significant difference (ANOVA) between the scores of different sources of conflict, indicating that all of them were possible contributing factors in school conflict: interpersonal conflict (3.23); structural organizational weakness (2.99); poor organizational communication (2.88); limited availability of resources (2.84); leadership style (2.61); introduction of changes and innovations (2.48).

### 3.4. Conflict Management Styles

**Figure 4:**
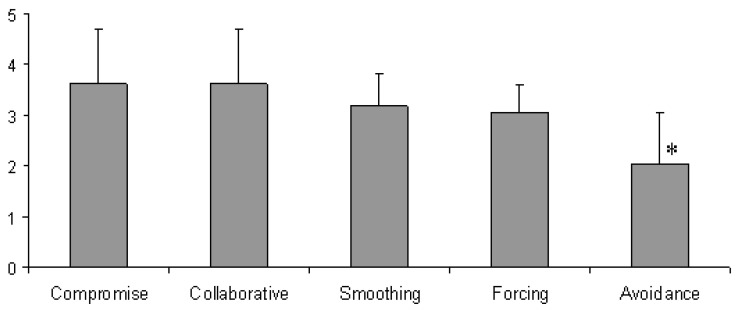
Teachers’ perceptions of their school leader’s conflict management style.

Teachers’ perceptions of their school leader’s conflict management style was assessed according to the scores of their answers (0 = never; 1 = rarely; 2 = sometimes, 3 = often; 4 = frequently) for different conflict management styles. A score above 3 indicates that a particular conflict management style was *frequently* used by school leaders, a score between 2 and 3 indicates that a particular conflict management was not *frequently* used by school leaders (Figure 4).

*Compromise* (3.63) and *Collaborative (3.63)* styles were *often/frequently* used, followed by *Smoothing (3.20), Forcing (3.05),* and *Avoidance* (2.05), which were *often* and *sometimes* exhibited by school leaders. Statistical analysis (Tukey’s HSD post-hoc tests, *p* < 0.05) indicated that, compared to the other conflict management styles, *Avoidance* exhibited significantly lower scores, indicating that this conflict management style was less frequently exhibited by school leaders.

**Figure 5:**
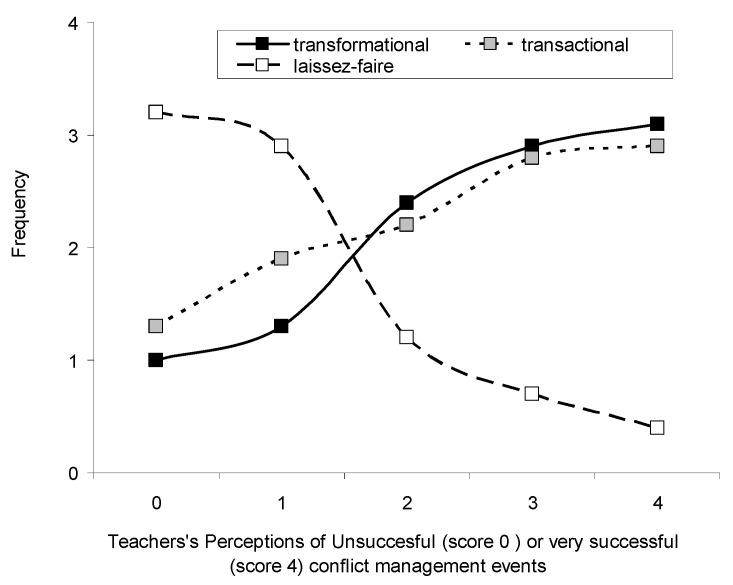
Perceived Leadership style and likelihood of successful resolution of school conflicts according to teachers. Scores range from 0 (unsuccessful) to 4 (successful). Lines represent different leadership styles: Transformational (solid line with black squares), transactional (dotted line with grey squares), laissez-faire (dashed line with white squares) and the effectiveness of their leader in handling school conflicts.

Teachers’ perceptions on the effectiveness of different leadership styles was assessed according to the scores of their answers for the successful resolution of school conflicts and the leadership style of their school leader. Scores ranged from 0 (unsuccessful) to 4 (very successful). The results are graphically presented in Figure 5. A score above 3 indicates that a particular leadership style was *often/frequently* associated with successful conflict resolution. Statistical analysis (ANOVA, *p* < 0.05) indicated that transformational and transactional leadership exhibited an effect on conflict resolution, with equally higher effectiveness in *successful* (indicated by scores > 3) conflict resolution compared to laissez-faire leadership style (Tukey’s HSD post-hoc tests, *p* > 0.01), which was reported as *less effective* in conflict management.

**Figure 6:**
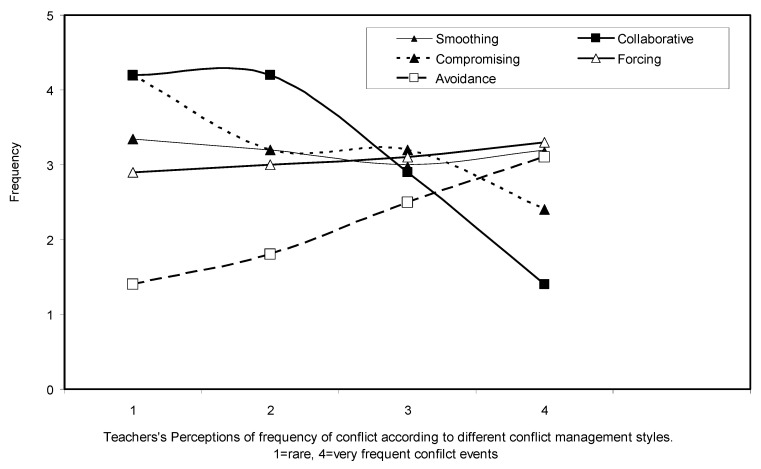
Conflict management style and frequency of school conflicts according to teachers’ perceptions. Lines represent different conflict management styles: *Smoothing* (solid line with black triangles), *Compromising* (dotted line with black triangles), *Collaborative* (solid line with black squares), *Avoidance* (solid line with white squares), *Forcing* (solid line with white triangles). Scores range from 1 (rare conflicts) to 4 (very frequent occurrences of school conflicts).

Teachers’ perceptions on the effectiveness of different conflict management styles was assessed according to the scores of their answers for the frequency of conflicts in their school and the conflict management style of their school leader. Scores ranged from 1 (*rare/low* frequency of conflicts) to 4 (*very frequent* occurrences of school conflicts). The results are graphically presented in Figure 6. A score above 3 indicates that a particular conflict management style was *often/frequently* associated with an increased likelihood of conflicts. Statistical analysis (ANOVA) indicated that *Smoothing* and *Forcing* had no effect on the likelihood of conflict occurring in a school unit. *Compromising* and *Collaborative* conflict management styles had a significant effect (Tukey’s HSD post-hoc tests, *p* > 0.05) with a reduced frequency of conflicts, whereas *Avoidance* was associated with a significantly increased frequency of conflicts (Tukey’s HSD post-hoc tests, *p* > 0.05). There was no significant difference between the scores of the transformational and transactional leadership styles, whereas the laissez-faire leadership style was significantly less frequently exhibited (Tukey’s HSD post-hoc tests, *p* < 0.01).

## 4. Discussion

The results of the present work support the hypothesis that the effectiveness of school conflict management may vary according to leadership style and conflict management style. The perceptions of teachers indicate that school conflict is a frequent issue and that a range of leadership styles and conflict management styles may be exhibited by School Principals. 

Greece has a highly centralized educational system with several sources of school conflicts often being generated by the centralized top-down decision and communication system. There is a limited number of similar research works for Secondary Education School Units in the region of Epirus in Greece.

The results indicate that conflict is a frequent problem, and conflict frequency and conflict resolution may vary. The results provide some evidence about the significant role of school leaders and on the effectiveness of school leaders’ conflict management in Greece and can be used to identify possible weaknesses in the current settings in respect to conflict management. 

Leadership and conflict management styles are contributing parameters for the effectiveness of any organization. Effective leadership is crucial for the overall success of any organization, ensuring its growth and long term performance. 

Competent leaders should lead, inspire and identify the appropriate conflict management strategy as well as the conflict management qualities and skills or solution strategies most suitable for each situation [35]. In the same manner, school leaders have to decide how to address school conflicts using different strategies, as discussed by Saiti [24].

### 4.1. Leadership Style

The teachers who participated in the present research work considered transformational and transactional leadership as being most frequently exhibited by their school leaders, whereas laissez-faire was significantly less frequently exhibited (Figure 1). This is in agreement with other studies in Greece that reported similar results [17,18]. In fact, transformational and transactional leadership styles may coexist, with school leaders adopting their style according to the particular situations they encounter [27]. 

In the present study, the perceived leadership style varied according to school size, with transformational leadership being exhibited in larger schools, whereas the laissez-faire leadership style was exhibited more frequently in small schools. Transactional leadership style was equally frequently used in the small and large school units. 

There is some evidence to suggest that school size may affect leadership style [33]. Contributing factors for this size effect may be: differences between urban and suburban external environments, school size, teachers’ perceptions and values [36].

Environmental factors, such as school size or school culture, can contribute to the leadership style differences observed in the present work [37]. For example, school climate may vary according to school size [38], in turn, school climate and leadership style may change according to school size [39]; transformational leadership is found to be associated with a constructive school culture, whereas transactional leadership is associated with a defensive culture [40,41].

### 4.2. Frequency of School Conflicts

Conflict appeared to be a frequent issue concerning the schools in the present work (Figure 2). Among the frequent sources of conflict were identified: interpersonal conflict; structural organizational weakness; poor organizational communication; limited availability of resources; leadership style and introduction of changes and innovations. These results are in agreement with previously published reports on the contribution of personal and organizational factors for school conflicts in Greece and other countries [24,42,43].

### 4.3. Conflict Management Styles

According to the perceptions of the teachers’ who participated in the present work, their school leaders exhibited a range of conflict management styles. *Compromise* and *Collaborative* styles were frequently observed, followed by *Smoothing* and *Forcing*. *Avoidance* was less frequently exhibited by school leaders. *Compromising* and *Collaborative* styles were included in the prevailing conflict management styles exhibited by school leaders, contributing to creating a constructive and effective conflict management style adopted by school leaders in Greece [24]. *Forcing* and *Avoidance* may have undesired effects on school functions and climate and are therefore less popular [42].

A negative effect of *Avoidance* was also exhibited in the effectiveness of conflict management in the present work. Teachers perceived *Avoidance* as the least effective conflict management style, providing further justification for the low popularity of this conflict management style observed in the present work.

### 4.4. Effectiveness of the School Leaders in Conflict Management

Apart from the differences in conflict management style, differences were also observed in teachers’ perceptions about the frequency of conflicts and the leadership styles of Principals in their schools. The results of the present work indicate that the school Principals’ leadership style had significant influence on effective conflict management. 

Transformational and transactional leadership styles were perceived by teachers as equally effective in successful conflict resolution, whereas the laissez-faire leadership style was not. These results are in agreement with previous reports on the inferiority of the laissez-faire leadership style and the expected benefits of transformational leadership on effective school management [16,17,18]. 

Leaders set the tone for conflict management through their leadership styles. Principals’ work experience and how they perceive power both influence their strategies during conflict management.

Experienced transformational school Principals choose conflict management strategies that ensure relationships are not destroyed and that relations with other members of staff or the school community involved in conflict management do not get affected negatively [33]. 

Transformational leadership is popular and frequently adopted by school leaders in Greece. The benefits of this style include innovation and creativity. Transformational school leaders can create a synergistic environment that motivates and enhances collaboration towards change. A transformational leader can help conflicting groups work together towards their common goals by providing encouragement and support, creating an environment that releases tensions, and professionally handles disruptive behavior. 

School Principals who perceive themselves as transformational leaders prefer collaboration as the best and most frequent strategy of conflict management. Transformational leadership tends to encourage flexibility and creativity with an emphasis on critically questioning the set policies, evaluating strategies, and gaining effective performance [44]. 

Transactional leadership is also a frequently observed leadership style in Greek schools. This style can be effective in a highly centralized setting as in the case of the educational system in Greece. Transactional leaders can be successful in maintaining consistency with a plethora of national laws, circulars, and guidelines given by the Ministry of Education. An effective leader can identify the conflict management strategy that is appropriate for each case and recognize which conflict management qualities and skills or solution strategy is most suitable for each situation [32].

Leadership style can affect school innovation and pupils’ performance [45], school climate, and teachers’ satisfaction [12] and consequently has direct and indirect effects on the sources of school conflicts.

### 4.5. Practical Recommendations

There are several challenges that school leaders in Greece face. Training in human resources management skills may have potential benefits on several parameters of effective school management, including motivation and job satisfaction, students’ success, team building, and commitment [46,47,48,49,50,51,52,53]. 

## 5. Conclusions

The results of the present work indicate that leadership and conflict management style was associated with the effectiveness of conflict management. The sample reflected a range of leadership and conflict management styles but it is difficult to derive causal relationships from a cross-sectional study. Further investigation is required to confirm a possible direct or indirect effect of leadership and conflict management styles on effective conflict management. 

In-service or pre-service training of school Principals in human resources management skills, including leadership and conflict management, can aid in improving the efficiency of leaders in Greek schools. The expected benefits of effective conflict management extend to other interacting parameters. Effective conflict management can create conditions that will improve school climate, teachers’ job satisfaction and commitment and school performance. 

## Figures and Tables

**Table 1:** Demographic characteristics of the teachers (n = 128) who participated in the current survey.

Characteristic	n	Percentage %
Age		
≤ 30	3	2.3
30–40	33	25.8
40–50	57	44.5
> 50	35	27.3
Gender		
♀	73	57
♂	55	43
Work Experience		
≤ 5	8	6.3
5–15	58	45.3
15–25	47	36.7
> 25	15	11.7
Years serving in the current school unit		
≤ 5	70	54.7
5–15	46	35.9
15–25	8	6.3

**Table 2:** Multifactor Leadership Questionnaire (MLQ) factors of teachers’ perceptions of their School Principal’s leadership characteristics. Significant differences (Tukey’s honestly significant difference (HSD) post-hoc tests, *p* < 0.05) between the scores of different leadership characteristics are indicated with at least one different letter.

Leadership Characteristics		Mean	Cronbach’s α
*Transformational characteristics*	Idealized influence (attributed)	3.54 ± 1.11a	0.88
	Idealized influence (behavior)	3.41 ± 0.87a	0.66
	Inspirational motivation	3.38 ± 1.00a	0.88
	Intellectual stimulation	3.31 ± 1.01a	0.85
	Individualized consideration	3.05 ± 0.91a	0.64
*Transactional characteristics*	Contingent reward	3.55 ± 0.90a	0.77
	Management-by-exception (active)	3.22 ± 0.65a	0.68
	Management-by-exception (passive)	2.63 ± 1.01bc	0.80
	Laissez-faire leadership	2.00 ± 1.07c	0.88

**Table 3:** Comparison of leadership style exhibited in small school units (≤10 teachers serving, n = 7) and large school units (>30 teachers serving, n = 3).

	Small School Units	Large School Units	Tuckey’s Test
*Transformational*	2.69 ± 1.21	3.39 ± 0.77	*p* = 0.039
*Transactional*	3.07 ± 0.53	3.39 ± 0.66	NS *p* = 0.227
*Laissez-faire*	3.11 ± 1.11	2.28 ± 0.94	*p* = 0.035
